# Metochalcone induces senescence-associated secretory phenotype via JAK2/STAT3 pathway in breast cancer

**DOI:** 10.32604/or.2023.044775

**Published:** 2024-04-23

**Authors:** JIANBO ZHOU, FENG WAN, BIN XIAO, XIN LI, CHENG PENG, FU PENG

**Affiliations:** 1 Department of Pharmacology, West China School of Pharmacy, Sichuan University, Chengdu, China; 2 State Key Laboratory of Southwestern Chinese Medicine Resources, Chengdu University of Traditional Chinese Medicine, Chengdu, China; 3 Key Laboratory of Drug-Targeting and Drug Delivery System of the Education Ministry, Sichuan Engineering Laboratory for Plant-Sourced Drug and Sichuan Research Center for Drug Precision Industrial Technology, Sichuan University, Chengdu, China; 4 Chengdu No. 1 Pharmaceutical Co., Ltd., Pengzhou, China; 5 Chengdu Push Bio-Technology Co., Ltd., Chengdu, China

**Keywords:** Metochalcone, Breast cancer, Lung cancer, SASP, JAK2/STAT3

## Abstract

Breast and lung cancers are the leading causes of mortality and most frequently diagnosed cancers in women and men, respectively, worldwide. Although the antitumor activity of chalcones has been extensively studied, the molecular mechanisms of isoliquiritigenin analog 2', 4', 4-trihydroxychalcone (metochalcone; TEC) against carcinomas remain less well understood. In this study, we found that TEC inhibited cell proliferation of breast cancer BT549 cells and lung cancer A549 cells in a concentration-dependent manner. TEC induced cell cycle arrest in the S-phase, cell migration inhibition *in vitro*, and reduced tumor growth *in vivo*. Moreover, transcriptomic analysis revealed that TEC modulated the activity of the JAK2/STAT3 and P53 pathways. TEC triggered the senescence-associated secretory phenotype (SASP) by repressing the JAK2/STAT3 axis. The mechanism of metochalcone against breast cancer depended on the induction of SASP via deactivation of the JAK2/STAT3 pathway, highlighting the potential of chalcone in senescence-inducing therapy against carcinomas.

## Introduction

**Figure 1:**
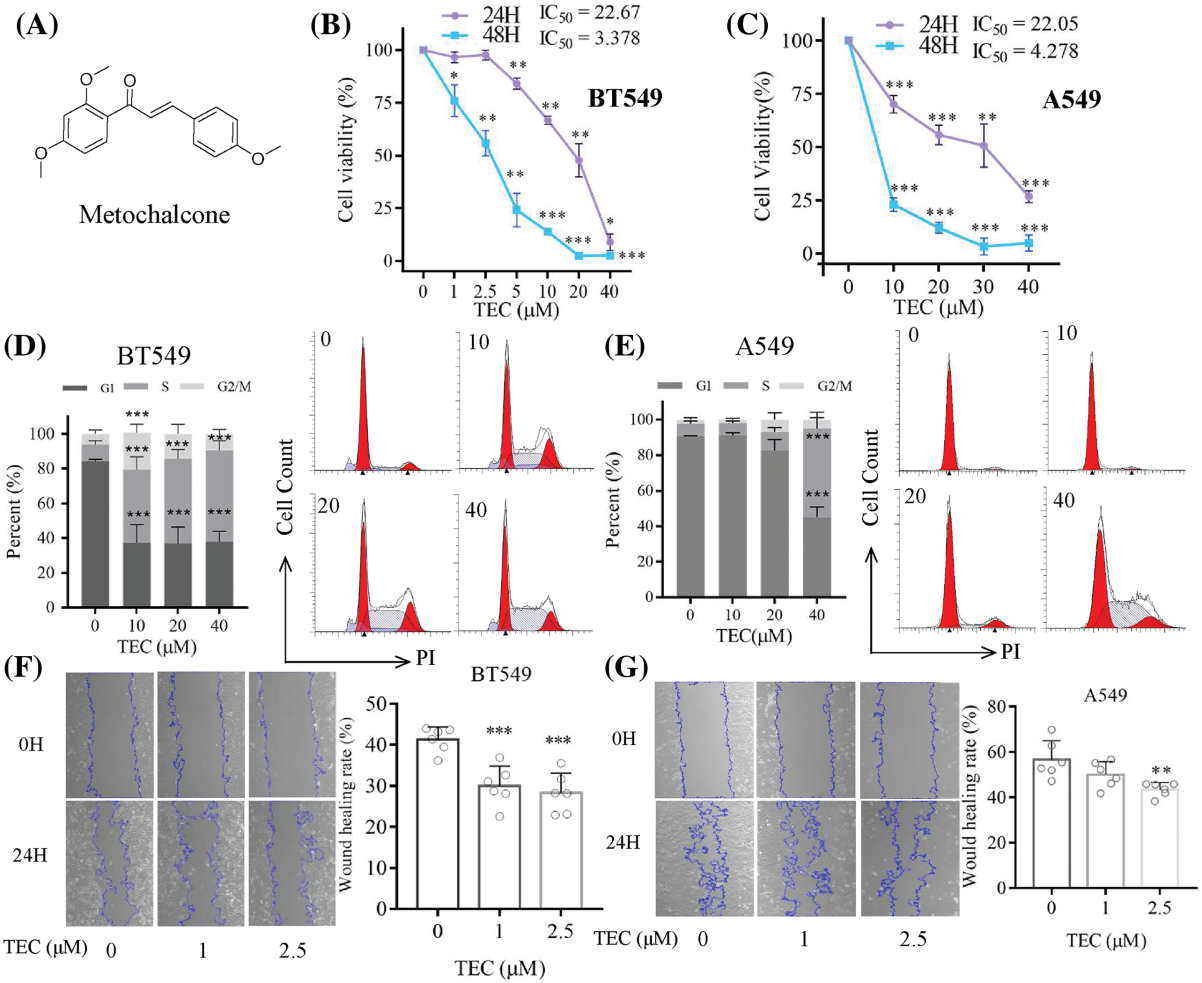
TEC inhibited cell proliferation and migration, and reduced cell cycle arrest in BT549 and A549 cells. (A) The molecular structure of TEC (PubChem CID: 6063342). (B and C) The CCK-8 method showed TEC inhibited proliferation at 24 h and 48 h. (D and E) TEC induced cell cycle arrest in BT549 and A549 cells at 24 h. (F and G) TEC suppressed cell migration in wound healing assays at low concentrations (200×). *p < 0.05; **p < 0.01; ***p < 0.001.

Breast and lung cancers have the highest mortality and diagnosis rates in women and men, respectively [1,2]. Traditional Chinese medicine and ethnopharmacology have considerable potential as molecular libraries for screening small molecules against cancers [3]. Although traditional Chinese medicine is considered a complementary and alternative treatment for cancer treatment [4], Caulis Spatholobi (the stem of *Spatholobus suberectus* Dunn), a traditional Chinese medicine to potentialize blood circulation and eliminate stasis [5], remains poorly understood. The efficacy of Caulis Spatholobi against tumor metastasis in colorectal and breast cancers has been demonstrated through alleviating tumor cell-induced platelet aggregation and reshaping epithelial-mesenchymal transition in preclinical studies [6,7]. Several flavonoids, including chalcones, have been identified as the bioactive components in Caulis Spatholobi [8]. In a previous study on *Spatholobus suberectus* Dunn, we reported the anti-proliferative properties of 2', 4, 4'-trimethoxychalcone (TEC, Fig. 1A) or metochalcone against breast cancer [9].

Cellular senescence is a common and inevitable physiological process in cells, including cancer cells, featured by cell cycle arrest, an increase in senescence-associated β-galactosidase, structural and metabolic changes, and so on [10]. The senescence-associated secretory phenotype (SASP) has all the hallmarks of cell senescence and is characterized by the numerous secretions of pro-inflammatory cytokines, growth factors, and so forth. SASP is initiated by several signaling pathways, mainly NF-κB, CEBPβ, JAK/STAT, p38 MAPK37 and mTOR, etc [11]. Targeting senescent cells and therapy-induced SASP are viewed as strategies against carcinomas [10]. Aberrant activation of the JAK2/STAT3 signaling pathway deteriorates cancer progression and induces an immunosuppressive tumor microenvironment [12]. One of the most common mechanisms of traditional Chinese medicine and natural products against breast cancer is modulation of the JAK2/STAT3 pathway [13]. JAK2/STAT3 inhibition facilitates chemotherapeutic response by remodeling SASP [14], but whether small molecules induce SASP by inhibiting STAT3 signaling remains unknown.

Here, we focused on high-risk triple negative breast cancer and lung cancer to further investigate the cytotoxic effects and molecular mechanisms of TEC in breast cancer cell line BT549 and lung cancer A549 cells. First, TEC inhibited proliferation, cell cycle arrest, and migration in a concentration-dependent manner, and decreased the phosphorylation of JAK2/STAT3 and total JAK2/STAT3 expression in both cell lines. Pathway enrichment analysis implied that TEC induced senescence-associated secretory phenotype (SASP) in BT549 cells, which was identified by increasing β-galactosidase staining, senescence-associated secretory factors: IL-6, IL8, TGF-β1, and SASP markers: P16, P21, P53. Mechanistically, TEC inactivated the JAK2/STAT3 signaling axis and relieved its inhibitory effect on SASP. Our results unveiled the molecular mechanisms of metochalcone against carcinomas, which are dependent on the JAK2/STAT3 signaling axis to induce SASP. This makes TEC a promising candidate for senescence-induced cancer therapies.

## Materials and Methods

### Cell culture and cell viability assay

The A549 and BT549 cell lines were provided by the Cell Bank of the Chinese Academy of Sciences (Shanghai, China) and were cultured in RPMI 1640 medium (C11995500bt, Gibco, Beijing, China) with 10% fetal bovine serum (Gibco; Thermo Fisher Scientific, Waltham, MA, USA) and 1% penicillin and streptomycin solution (SV30010, HyClone, Logan, UT, USA) at 37°C and 5% CO_2_. Cells were seeded into 96-well plates (5,000 cells/well) for the cell counting kit-8 (CCK-8) cell proliferation assay. After treatment with TEC (Synthesized by WuXi AppTec, >98% content confirmed by HPLC) at the indicated concentrations for 24 or 48 h, the CCK-8 agent (CK04, Dojindo, Kumamoto, Japan) was added and incubated for 1–2 h, and the absorbance was then measured using the microplate reader (Varioskan Flash, Thermo Fisher Scientific, Inc.) at 450 nm.

### ELISA and β-galactosidase staining

Enzyme-linked immunosorbent assay (ELISA) was performed to examine senescence-associated factors (IL-6, IL-8 and TGF-β1). Briefly, cells were seeded in the 96-well plates and incubated for the indicated time periods and drug concentrations. Valukine ELISA kits (IL-6, #VAL102; IL-8, #VAL103; TGF-β1, #VAL127) were purchased from R&D Systems, Inc (Minneapolis, MN, USA). and applied to quantify protein levels in supernatant according the manufacturer’s instructions. Cell senescence status was assessed using a β-galactosidase staining kit (C0605, Beyotime, Shanghai, China) following manufacturer’s instruction.

### Cell cycle, migration and invasion

Cells were seeded into a 6-well plate (5 × 10^5^ cells/well) and incubated with TEC for 24 h. Cells were fixed with 70% ethanol overnight at −20°C before incubation with propidium iodide cycle kit (Nanjing KeyGen Biotech. Co. Ltd., China) for cell cycle analysis. Flow cytometry analysis was exerted using flow cytometer (CytoFlex, Beckman Coulter, USA), and the MODIFIT LT 5.0 (BD Biosciences, USA) was used for data analysis. In wound healing assay, when the well was fully covered with cells, a 10 μL pipette tip was used to create scratches, and then drugs were added and incubated for 24 and 48 h. Photographs of scratches were taken for statistical analysis using Image J software (NIH, Bethesda, MD, USA). Cells (1 × 10^5^) were plated into the upper Transwell chamber (#3428, Corning Incorporated, NY, USA), and complete growth medium was added to the lower chamber. After 24 h of drug treatment in the upper chamber, cells were washed with PBS buffer, fixed with 4% paraformaldehyde, stained with 0.1% crystal violet (Sigma-Aldrich, V5265), and photographed under an inverted microscope. Corning® BioCoat® Matrigel® Invasion Chambers plate (#354480, Corning) was used for invasion analysis with consistent procedures. The migrated and invaded cells were counted using the ImageJ software.

### Western blot, RNA-sequencing and real-time PCR

Proteins were extracted from the cells using RIPA lysis buffer (Beyotime, P0013) and quantified using the bicinchoninic acid (BCA) protein assay kit (Beyotime, P0009). Denatured total proteins were loaded onto a 10% SDS-PAGE gel and then transferred onto a PVDF membrane. The blots were incubated with monoclonal antibodies (STAT3, 4904; p-STAT3(Tyr705), 9145; JAK2,3230; β-actin, 4970; P21, 2947; P53, 2527; Cell Signaling Technology, Inc., Danvers, MA, USA; P16 INK4A, 10883-1-AP, Proteintech Group, Inc., Wuhan, China), followed by incubation with secondary horseradish peroxidase (HRP)-conjugated anti-rabbit antibody (7074, CST). After washing, the membranes were subjected to chemiluminescence, and the relative optical density was analyzed using Image J software (NIH, Bethesda, MD, USA). After 24 h of drug incubation, cells were lysed with TRIzol® reagent (15596-018, Life Technologies, USA) for RNA extraction and sequencing service was provided by Biomarker Technologies (Beijing, China). The R software 4.0.4 was employed to identify differentially expressed genes (DEGs) through the limma package. The Gene Ontology (GO) annotation and Kyoto Encyclopedia of Genes and Genomes (KEGG) pathway enrichment were performed using the ClusterProfiler package. Pathway activity analysis of gene expression matrix was performed using the PROGENy method [15]. Cells (1 × 10^6^) were seeded into a culture dish and treated with drug for 24 h. Total RNA was extracted using the TRIzol regent. Reverse transcription was performed to synthesize cDNA using PrimeScript™ RT reagent kit (RR047A, Takara bio Inc., Shiga, Japan). Quantitative PCR analysis was performed using Quantum Studio 3 (Thermo Fisher Scientific, Waltham, MA, USA) based on the SYBR kit TB Green® Premix Ex Taq™ II (Takara, RR820A). The thermal cycling conditions were set according to the manufacturer’s instructions, and the primer sequences were as follows:

STAT3, forward primer: CACCTTTGACATGGAGTTGACC, reverse primer: AGCAGATCACCCACATTCACT; GAPDH, forward primer: AATGGGCAGCCGTTAGGAAA, reverse primer: GCCCAATACGACCAAATCAGAG. The relative expression of mRNA levels was measured via the 2^−ΔΔCT^ method.

### Animal experiments

BALB/c nude mice were purchased from SPF Biotechnology Co., Ltd. (Beijing, China). Animal experiments were performed at the Animal Central of Chengdu University of Traditional Chinese Medicine (Approved ID: 2023004). This was approved by our Animal Ethics Committee. Male and female mice were subcutaneously injected with A549 or BT549 cells (5 × 10^6^ cells/mouse) to establish a cell-derived xenograft model, respectively. Animals were caged in a specific pathogen-free laboratory with free access to food and water. When the volume of the tumor was greater than 50 mm^3^, animals were grouped. Subsequently, they were given an intraperitoneal injection of drugs (40 mg/kg/day) or an equal volume of solvent (control group) for 21 days. After the animals were euthanized by cervical dislocation, the body weight, tumor weight, and tumor volume were measured.

### HE and IHC

After tumor tissues were formalin-fixed and paraffin-embedded, they were sliced into 4-μm thick sections for HE (hematoxylin-eosin) staining and Immunohistochemical staining (IHC) for Ki67 (GB111499,1:200, Servicebio, Wuhan, China), P16-INK4A (10883-1-AP, 1:100, Proteintech, Wuhan, China) and P21 (YT3794, 1:50, Immunoway, Jiangsu, China). HE staining was performed using HE staining kit (C0105S, Beyotime). Besides, sections were subjected to deparaffinization, rehydration and antigen retrieval. After blocking, these samples were incubated with primary antibody overnight at 4°C. Subsequently, the slides were covered with HRP-conjugated secondary antibody goat anti-rabbit IgG(H+L) HRP (GAR0072, 1:200, Liankebio, Huangzhou, China) or goat anti-mouse IgG(H+L) (bs-40296G-HRP, 1:200, Bioss, Beijing, China) and then stained with diaminobenzidine (DAB) chromogen kit. Images were captured using an inverted microscope (Eclipse Ci-L, Nikon, Japan). The staining results were analyzed using Image-pro Plus 6.0 (Media Cybernetics, Rockville, MD, USA) and quantified by integrated optical density (IOD).

### Molecular docking and microscale thermophoresis

Molecular docking was performed using AutoDock 4 and AutoDock Vina with the blind docking, and the structure of TEC and 21 STAT3 inhibitors were retrieved from PubChem (https://pubchem.ncbi.nlm.nih.gov/). The 3D protein structures of the STAT3 monomer (PDB ID: 6njs) and aggregate (PDB ID: 6tlc) were downloaded from RSCB Protein Data Bank (https://www.rcsb.org/), and then processed using PyMol and AutoDockTools. The docking results were visualized by Pymol to display the polar contacts. The affinity heatmap was generated by the Heatmap package in R studio software. The microscale thermophoresis (MST) assay was used to detect the direct affinity between the small molecule and protein *in vitro*. STAT3 protein was purchased from ACROBiosystems (ST3-H5149, ACROBiosystems, Newark, DE 19711, USA) and labeled using the His-Tag Labeling Kit RED-tris-NTA 2nd Generation (MO-L018, NanoTemper Technologies, Munich, Germany). MST analysis was performed in the Monolith NT.115 system (NanoTemper Technologies) according to the manufacturer’s manual. Results were normalized and analyzed in the Monolith NT.115 system.

### Statistical analysis

GraphPad Prism software (version 9.0) was applied to calculate the significance of all experiments that were repeated three times in parallel. Student’s *t*-test was used to determine significance between two groups, and one-way analysis of variance (ANOVA) was used for multiple groups. The threshold of statistical significance was set at *p* value < 0.05 (ns, *p* ≥ 0.05; *p < 0.05; **p < 0.01; ***p < 0.001; ^#^*p* < 0.0001).

## Results

### TEC inhibits cell proliferation, cell cycle, migration and invasion

**Figure 2:**
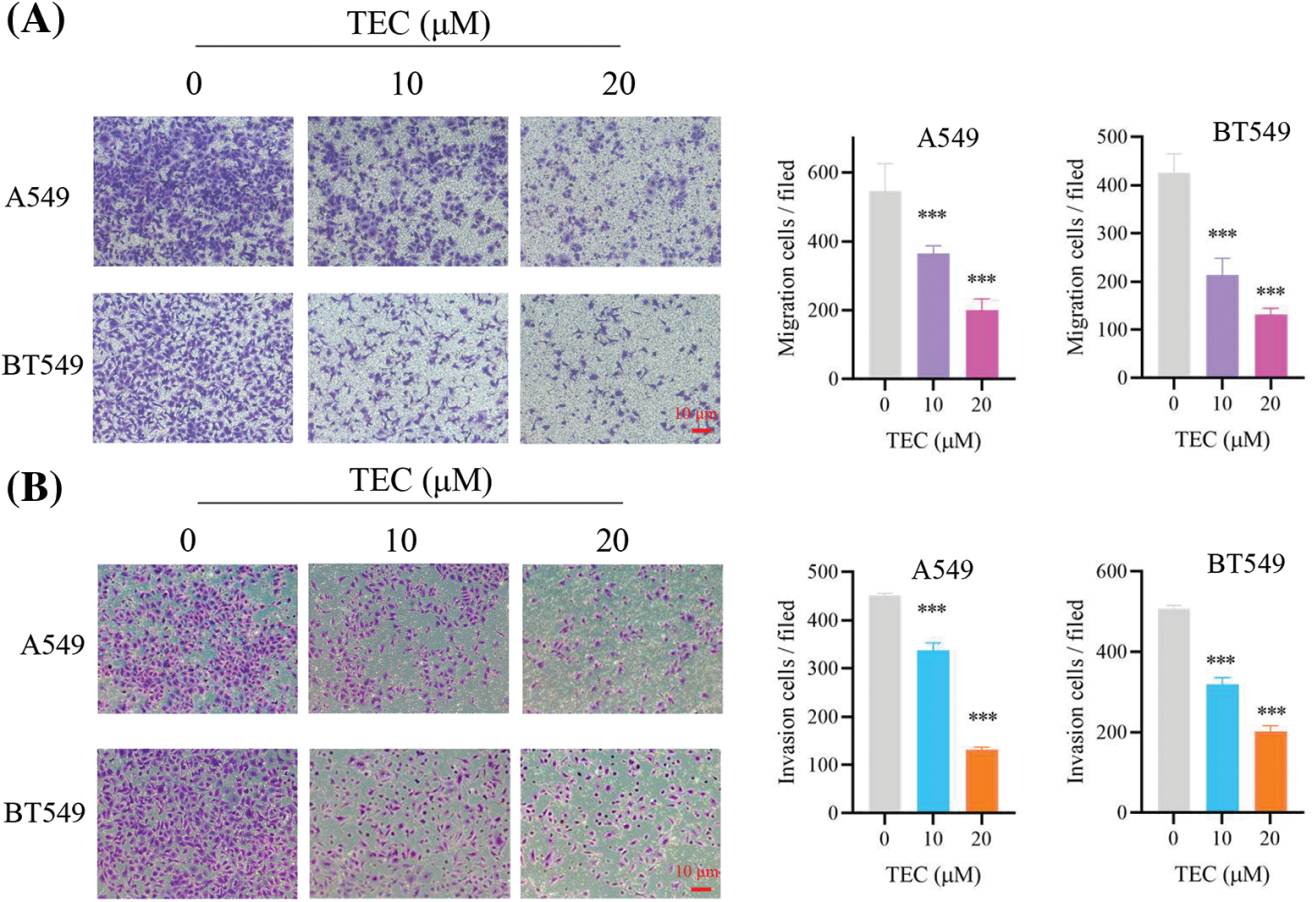
TEC inhibited migration (A), invasion (B) in BT549 and A549 cells. The red scale bar represents 10 μM. ***p < 0.001.

First of all, TEC inhibited cancer cell proliferation with time- and concentration-dependent (Figs. 1B and 1C). The IC_50_ values of BT549 and A549 at 24 h were 22.67 and 22.05 μM, respectively. After TEC treatment at 48 h, the IC_50_ values of BT549 and A549 were low at 3.378 and 4.278 μM, separately. Cell cycle assays indicated that TEC induced the S-phase cell cycle arrest in both BT549 and A549 cells. Compared to A549 cells, better significant outcomes were observed in BT549 cells at concentrations including 10, 20, and 40 μM (Figs. 1D and 1E). In wound healing assay, TEC also suppressed cell migration in the above two cell types, which was more significant and effective in BT549 cells (Figs. 1F and 1G). The Transwell assays revealed that TEC emasculated cancer cell migration and invasion *in vitro* (Fig. 2).

### Potential TEC-regulated pathways revealed by transcriptomic analysis

**Figure 3:**
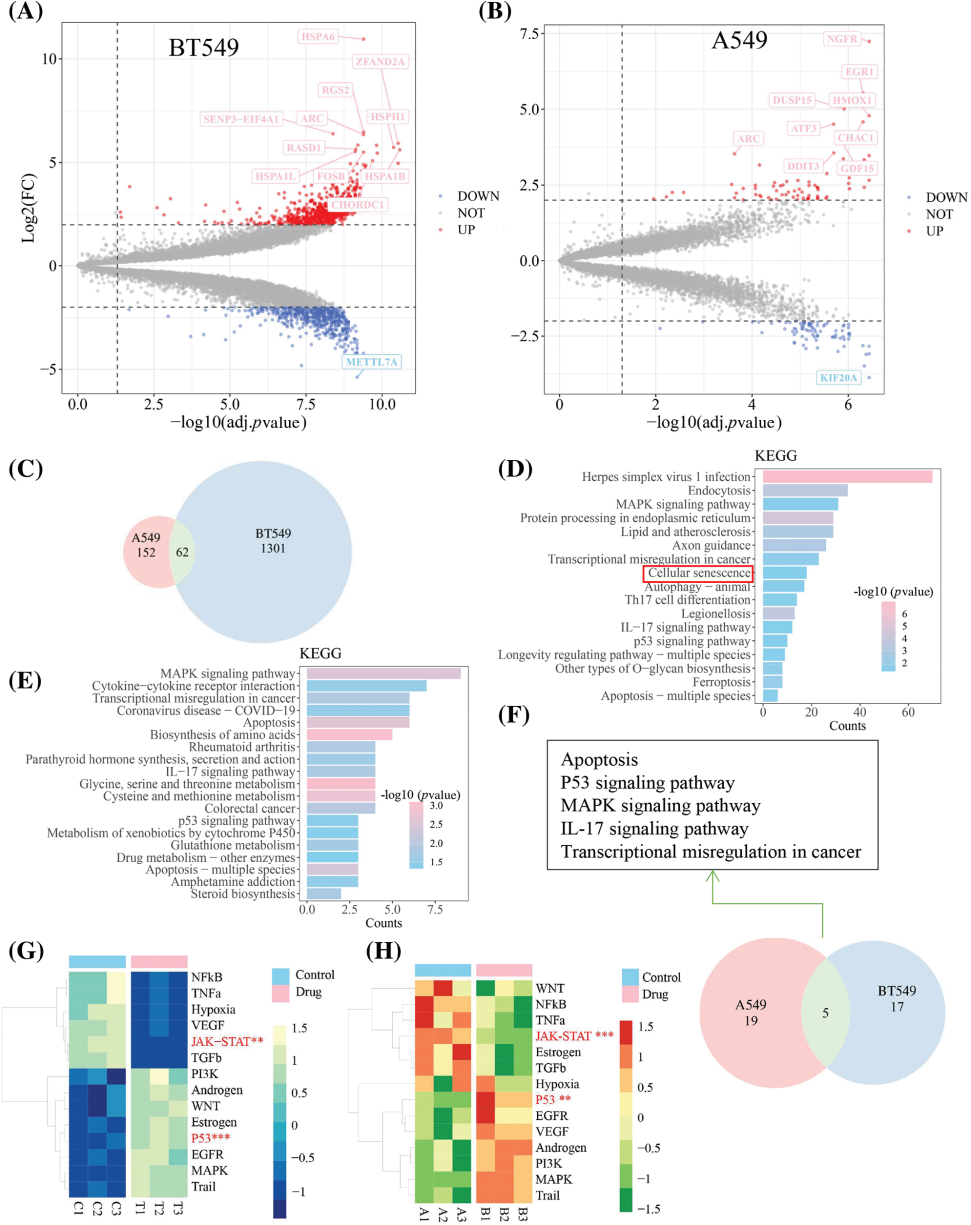
The results of RNA-sequencing in BT549 and A549 cells at TEC (20 μM) for 24 h treatment. (A and B) Volcano map of different expression genes (DEGs) in BT549 and A549 cells, with the threshold | log_2_(fold change) | > 2 and adjusted *p* value < 0.05. (C) Veen diagram showed the common DGEs. The Kyoto Encyclopedia of Genes and Genomes (KEGG) pathway analysis via R package clusterProlifer was implemented to the DGEs in BT549 (D) and A549 cell (E), respectively. Veen diagram implied common shared KEGG pathways between BT549 and A549 after TEC treatment. (F) Veen diagram implied common shared KEGG pathways. The pathway activity analysis with two-tail *t*-test of expression matrix in BT549 (G) and A549 cells (H), respectively. Color gradient in the heatmap represents the pathway activity score.

RNA-sequencing was performed to further investigate the underlying molecular mechanisms of TEC against breast and lung cancers. A total of 1301 differentially expressed genes (DEGs) were filtered with the threshold of *p* value < 0.05 and |log(fold change)| > 2, including 720 upregulated and 581 downregulated genes in BT549, while 152 different DEGs (72 upregulated and 80 downregulated genes) were screened in A549 cells with the consistent threshold (Figs. 3A and 3B). The 62 shared DEGs were identified using a Venn diagram (Fig. 3C).

The HSPA6, a heat shock protein family A (Hsp70) member 6, a tumor suppressor that inhibits proliferation, migration and invasion of BT549 cells, is regulated by Thymoquinone [16]. In this study, HSPA6 was found to be the most significantly upregulated gene in BT549 cells. Another putative TEC-targeted gene was METTL7A (methyltransferase like 7A), which was reported as a methyltransferase that was down-regulated by TEC in BT549 cells and was viewed as a tumor suppressor in breast cancer [17]. The NGFR, also known as p75 neurotrophin receptor (p75NTR) or CD271, is a number of the tumor necrosis factor receptor superfamily (TNFRSF) and induces apoptosis by increasing caspase activity [18]. A previous study has showed that the NGFR-AMPK-mTOR pathway was elevated by resveratrol to induce autophagy and apoptosis in A549 cells [19]. KIF20A, also known as mitotic kinesin-like protein 2 (MKLP2), plays an essential role in mitosis by serving as a kinesin to protect cells from chromosomal instability, and drives cytokinesis [20].

KEGG enrichment analysis was used to identify the enriched pathways of DEGs. KEGG results in BT549 cells were enriched in the well-known and universal pathways in cancer, including apoptosis, autophagy, ferroptosis, transcriptional dysregulation of MAPK, IL17 and P53 signaling pathways in cancer (Fig. 3D); the similar results, excluding autophagy and ferroptosis, were found in A549 cells (Fig. 3E). Notably, five shared pathways in both BT549 and A549 cells were responsible for the anti-tumoral mechanism, containing apoptosis, p53 signaling pathway, MAPK signaling pathway, IL-17 signaling pathway, and transcriptional dysregulation in cancer (Fig. 3F). Pathway activity analysis elucidated that TEC declined activity of the JAK/STAT3 and NF-κB signaling axes, while inducing activation of P53 pathway in both BT549 and A549 cells (Figs. 3G and 3H).

### TEC suppresses JAK2/STAT3 axis

**Figure 4:**
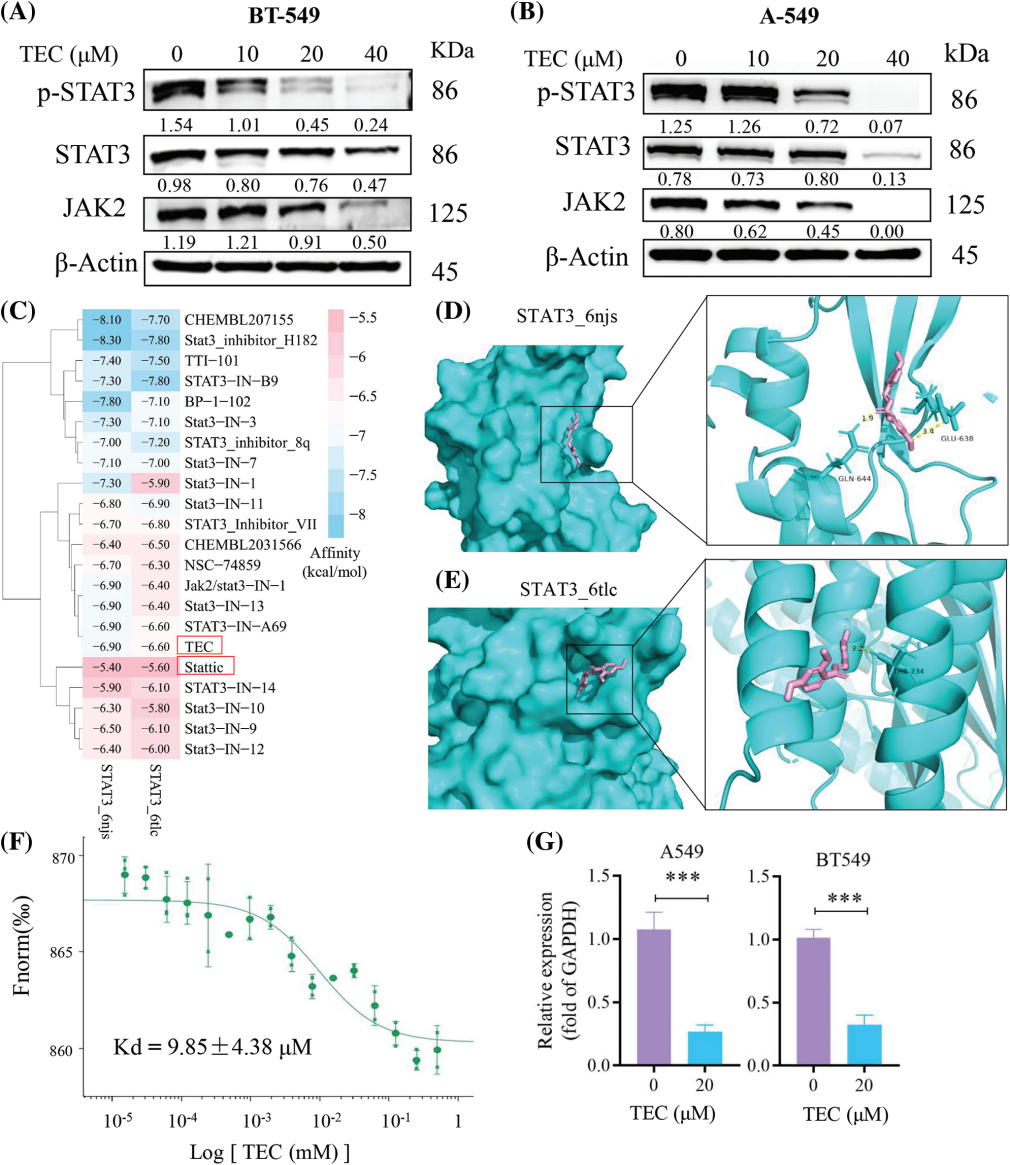
TEC inhibited JAK2/STAT3 signaling cascade. (A and B) Western blotting showed the protein expression after TEC treatment for 24 h in BT-549 and A549 cells. (C) The affinity heatmap between STAT3 proteins and STAT3 inhibitors were revealed by Autodock Vina. (D and E) The docking pattern between TEC (pink) and STAT3 proteins (cyan) was visualized by Pymol. (F) MST analysis revealed the affinity of TEC towards STAT3 protein (Kd = 9.85 ± 4.38 μM). (G) RT-PCR analysis of STAT3 mRNA expression with or without TEC treatment. ***p < 0.001.

The JAK2/STAT3 pathway plays an important role in the deterioration of breast cancer, including proliferation and metastasis [21]. Chalcone has been considered a potential molecular strategy for cancer prevention and treatment by targeting STAT3 [22]. Therefore, we evaluated the expression of important components of the JAK2/STAT3 pathway after TEC treatment for 24 h at indicated concentrations. As shown in Fig. 4A, TEC significantly reduced the protein expression of phosphorylated STAT3 and total JAK2/STAT3 in BT549 cells. However, TEC completely reduced the protein levels of p-STAT3 and JAK2 in A549 cells at a high concentration of 40 μM (Fig. 4B). Molecular docking was used to investigate TEC docking patterns with the STAT3 monomer (6 njs) and aggregate (6 tlc). TEC performed moderate affinity to STAT3 proteins among 21 small molecule STAT3 inhibitors (Fig. 4C), with affinities of −6.9 and −6.6 kcal/mol towards STAT3 monomer and aggregate, respectively. In molecular configuration, TEC interacted with the SH2 domain of STAT3 monomer via two hydrogen bonds (the methoxy of TEC linked with hydroxyl of glutamic acid-GLU638, the keto group of TEC connected with amido bond of glutamine-GLN644) (Fig. 4D). Consistently, STAT3 inhibitor SD-109 also bound the glutamine at the SH2 domain (glutamine-GLN644) of STAT3 [23]. The polar contact of TEC bound to the STAT3 aggregate was unmasked by the interaction of the methoxy group of TEC with the hydroxyl of threonine-THR234 (Fig. 4E). Furthermore, MST analysis confirmed TEC directly interacted with STAT3 protein with a dissociation constant (Kd) of 9.85 μM (Fig. 4F). Besides, TEC also reduced STAT3 mRNA expression (Fig. 4G).

### TEC induces senescence-associated secretory phenotype

**Figure 5:**
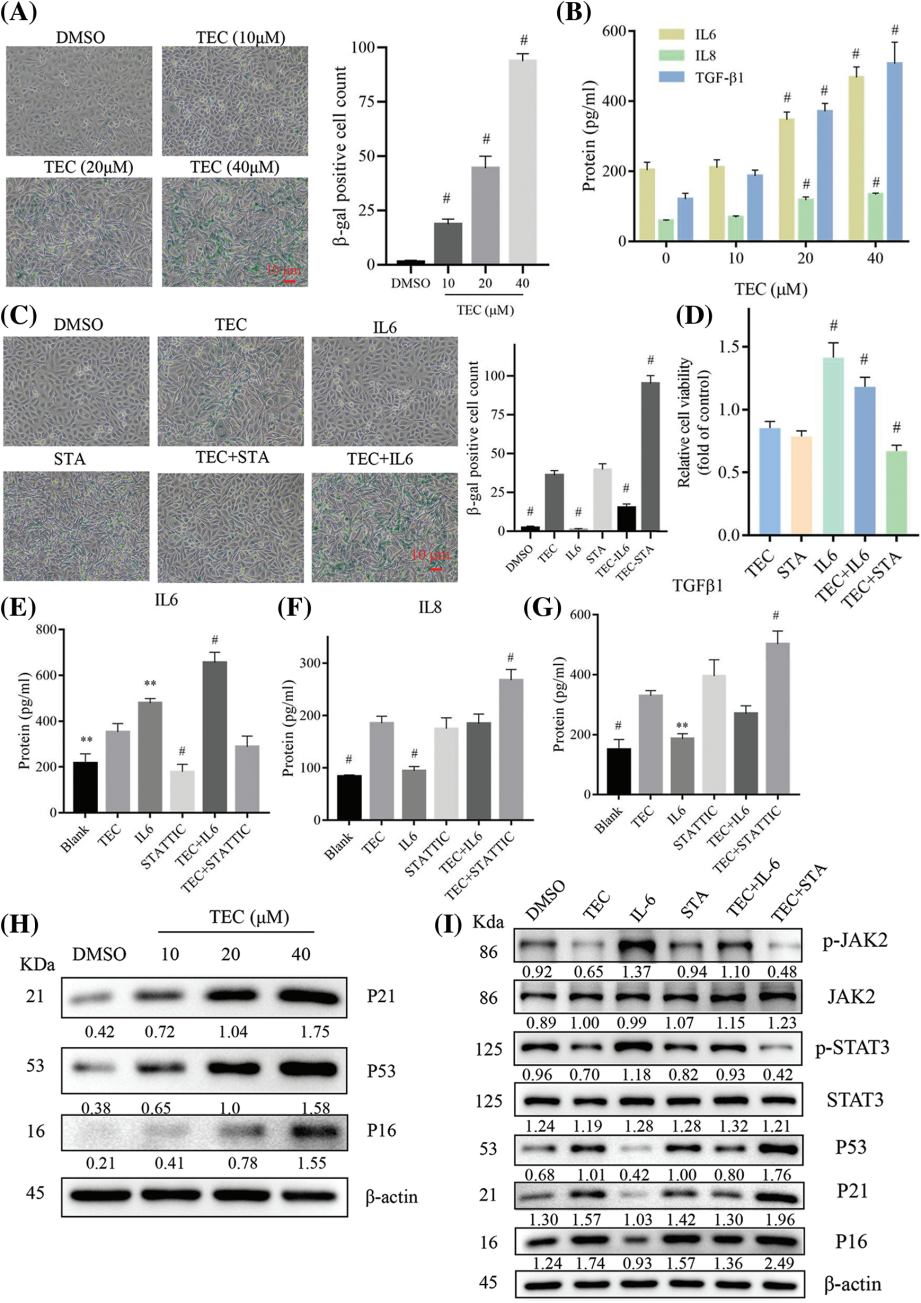
TEC induced senescence-associated secretory phenotype via JAK2/STAT3 pathway. (A) β-galactosidase staining (200×). (B) ELISA detected senescence associated secretions in cell supernatant after TEC treatment for 24 h. (C) β-galactosidase staining after TEC treatment (20 μM) with or without IL-6 (50 ug/mL) or STATTIC (STA, 2 μM), 200×. (D) Pharmacological intervention of the JAK2/STAT3 pathway altered the TEC-mediated proliferating-inhibition effect with or without IL-6 (50 ug/mL) or STATTIC (STA, 2 μM). (E–G) ELISA detected senescence associated secretions in cell supernatant after TEC treatment and/or pharmacological treatment. (H and I) TEC enhanced expression of SASP molecular markers: P16, P21, P53, and JAK2/STAT3 signaling axis. **p < 0.01; ^#^*p* < 0.0001.

KEGG pathway enrichment indicated that TEC induced SASP in BT549 cells, which was confirmed by the upregulated β-galactosidase activity with TEC concentration-dependent (Fig. 5A). Moreover, TEC also induced elevated levels of senescence-associated secretory factors: IL-6, IL8, TGF-β1, and increased protein expression of SASP-related markers: P16, P21, and P53 (Fig. 5B).

Furthermore, as shown in Fig. 5D, TEC-induced proliferating inhibition in BT549 cells was rescued by IL-6 (JAK2/STAT3 activator) and aggravated by STATTIC (JAK2/STAT3 inhibitor). The same trend that TEC-triggered cell senescence was reversed by IL-6 and exacerbated by STATTIC also was observed in β-galactosidase staining (Fig. 5C). Moreover, simultaneous treatment with TEC and STATTIC enhanced the secretory levels of SASP factors: IL8 and TGF-β1, while co-incubation with TEC and IL-6 reversed the TEC-mediated release of SASP factors (Figs. 5E–5G). Consistently, inhibition of JAK2/STAT3 cascade by STATTIC or TEC led to an increase in SASP markers, P16, P21, and P53, whereas the JAK2/STAT3 axis activated by IL-6 decreased SASP markers. Additionally, STATTIC increased TEC-induced SASP markers, whereas IL-6 declined the TEC-mediated effects of SASP induction (Figs. 5H and 5I).

### TEC inhibits tumor growth *in vivo*

**Figure 6:**
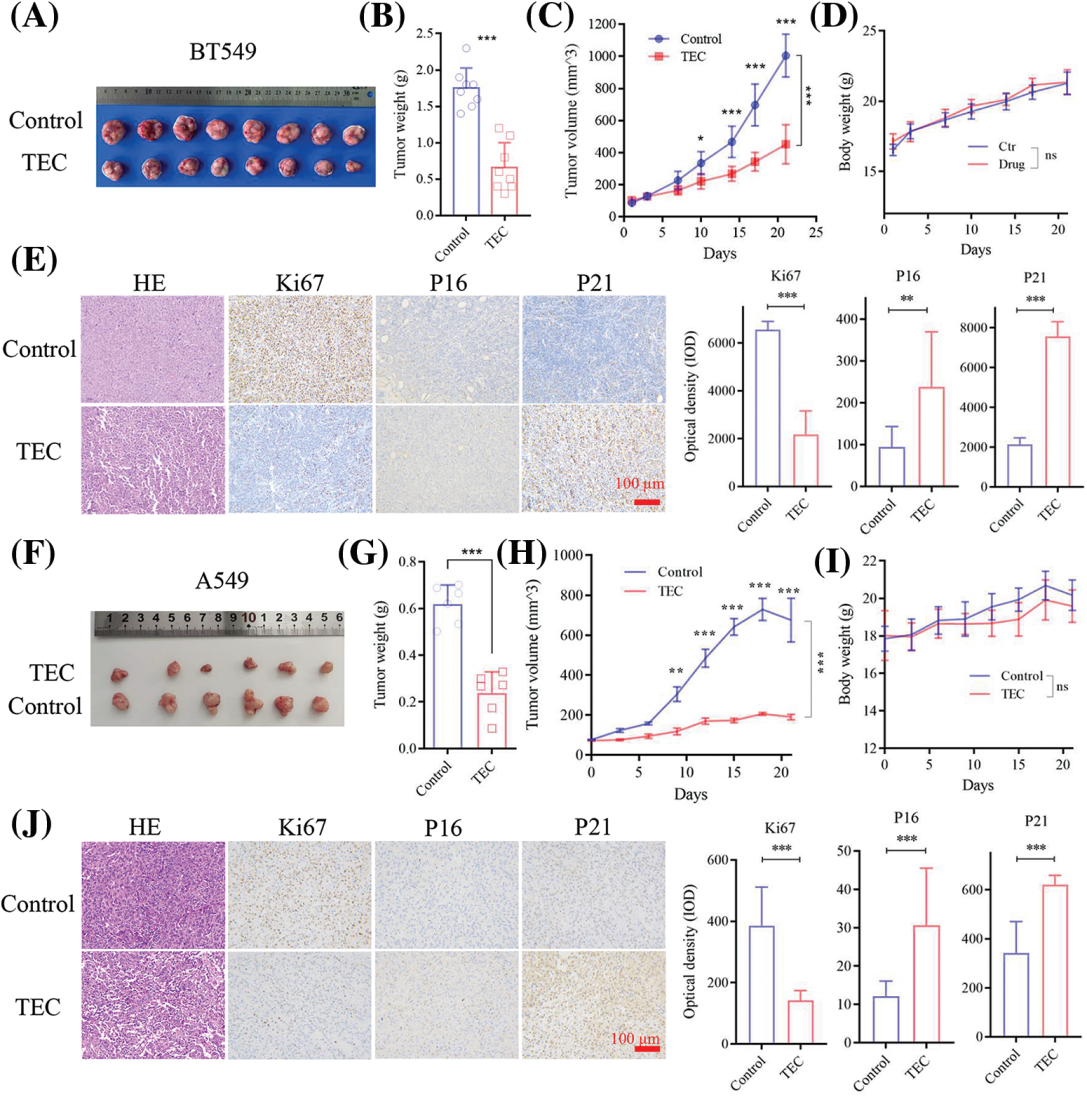
TEC reduced tumor growth in the BT549 cells xenograft mice model (A–E). (A) The tumor photo of the TEC (40 mg/kg) and control groups (8 mice per group). (B–D) The tumor weights, tumor volumes and body weight of mice in two groups. (E) HE and IHC staining of tumor tissue sections (Scale bar = 100 μm). TEC shrinks tumor growth in A549 cells-derived xenograft mice model (F–J). (F) The tumor photo of the TEC and control groups (6 mice per group). (G–I) The tumor weights, tumor volumes and body weight of mice in two groups. (J) HE and IHC staining of tumor tissue sections (Scale bar = 100 μm). ns, *p* ≥ 0.05; *p < 0.05; **p < 0.01; ***p < 0.001.

Further experiments in nude mice bearing xenograft tumors indicated that TEC inhibited tumor growth at a dose of 40 mg/kg/day, confirming the anti-tumor effects of TEC against BT549 and A549 cells *in vivo* (Figs. 6A and 6F). As shown in Figs. 6B and 6C, TEC significantly decreased the tumor weight and volume, compared to the solvent control group. No significant difference in body weight was observed between the two groups, indicating the safety of TEC *in vivo* (Fig. 6D). HE staining showed that TEC declined malignancy levels in tumors (decreased cell density and increased tissue spacing) (Fig. 6E). IHC staining revealed that TEC significantly reduced proliferation marker Ki67 and increased the expression levels of SASP markers P16 and P21 (Fig. 6E). These studies were also confirmed in the A549 cell-derived xenograft tumor model (Figs. 6F–6J).

## Discussion

Our study first demonstrated the anti-tumor effects of TEC against breast cancer and lung cancer via inhibition of the JAK2/STAT3 signaling and further revealed the potential role of metochalcone in restricting tumor growth *in vivo*. Transcriptional profiling revealed that TEC regulated latent and universal pathways, including apoptosis, P53 signaling pathway, MAPK signaling pathway, IL-17 signaling pathway, and transcriptional misregulation. Based on transcriptomic analysis, we expect to further investigate the molecular mechanisms of metochalcone that involve the NGFR-AMPK-mTOR pathway in lung cancer and METTL7A-mediated RNA m6a methylation and ferroptosis in breast cancer. Due to the high mortality and diagnosis rates in men (lung cancer) and women (breast cancer), we have chosen these two cancer cells for further experiments. Our cell bank includes BT549 breast cancer cells, A549 lung cancer cells, and MDA-MB-231 breast cancer cells. We will be using the MDA-MB-231 cell line to study TEC-induced ferroptosis, so only BT549 and A549 were chosen for this study.

Chalcones have emerged as potential molecular therapeutics against malignant carcinomas via modulation JAK2/STAT3 signaling pathway [22]. Various chalcone molecules targeting the JAK2/STAT3 axis, such as licochalcone, butein, isoliquiritigenin, 4,3′,4′,5′- tetramethoxychalcone [24], and Llicochalcone [25], have been utilized in cancer intervention. The anti-cancer properties of lutein (3,4,2′,4′-tetrahydroxychalcone) against various carcinomas involve the suppression of STAT3 activation in head and neck cancer, liver cancer, malignant pleural mesothelioma, myeloma, and prostate cancer [26,27]. In breast and lung cancers, butein also downregulates STAT3 phosphorylation [28]. In addition, isoliquiritigenin (ISL) [29,30], xanthoangelol, 4-hydroxyderricin [31], and xanthohumol contribute to the suppression of STAT3 expression in various cancer cells, including breast cancer, cholangiocarcinoma and pancreatic cancer [32].

Substantial evidence suggests that cell senescence plays a critical role in neoplasia, which is characterized by cell cycle arrest, and physiological and metabolic alterations. However, certain well-established features are commonly associated with the senescence phenotype, including activation of lysosomal function (increased activity of the lysosomal enzyme senescence-associated β-galactosidase), overexpression of cell cycle inhibitors P16 (INK4A), P21, P53 [33], and secretion of senescence-associated factors consisting of pro-inflammatory cytokines, matrix metalloproteinases, and growth factors [11]. P53, a senescence effector in the molecular context, contributes to cell cycle arrest and SASP expression, which depends on its role as a translational factor to increase SASP factor via activation of p38MAPK, NF-κB, C/EBPβ and so on [34]. Furthermore, the concept of “one-two punch” cancer therapy, combining pro-senescence therapy and senolytic therapy, has been considered an emerging and promising strategy to avoid paracrine and pro-oncogenic effects and to control neoplasia. This strategy has been realized in two programs: transformation of proliferating cells into senescent cells with pro-senescence treatment, followed by senolytic treatment to clean senescent cells [35]. Classically, SASP is induced and regulated through several pathways both in transcriptional and post-transcriptional modulation, such as NF-κB, JAK2/STAT3 signaling axis [34]. In particular, JAK2/STAT3 inhibition has been found to reprogram the SASP and improve the response to chemotherapy [14]. Two of the most extensively studied pro-inflammatory cytokines within the SASP are IL-6 and IL-8 [34]. Moreover, both anticancer and pro-tumor roles of TGF-β in cancer are contradictory, heterogeneous and molecular background-dependent [36]. Given the pivotal function of TGF-β in senescence re-enforcement and cancer immune evasion, it was selected as another SASP factor in this study [34,37]. Piperine, a natural bioactive component, modulated the secretion of SASP factors: IL-6, IL-8, and TGF-β1 [38]. Metformin alleviated the angiotensin II-induced SASP by down-regulating MMP-2, IL-6, and TGF-β [39]. In this study, TEC triggered SASP, as evidenced by increasing expression of P16, P21, and P53; elevated SASP factors (IL-6, IL-8, and TGF-β1); and activation of β-galactosidase. Of interest, KEGG pathway enrichment revealed the different expression genes in BT549 cells were enriched in cell senescence. Further results demonstrated that TEC-mediated SASP depended on the JAK2/STAT3 inhibition, rather than NF-κB signaling, and the activity of the JAK2/STAT3 pathway was reduced by TEC (activation of NF-κB drives SASP [11]). Although it is consistent with previous report that TEC-induced JAK2/STAT3 inhibition reprogramed SASP [14], it is still unclear how JAK2/STAT3 inhibition works and involves the crosstalk of SASP-relevant signaling pathway. While SASP reprograms the immune microenvironment with immunosupportive or immunosuppressive effects, it remains unclear whether TEC or TEC-induced SASP is involved in anti-tumor immunity [40] or the immune response based on PD-L1/PD1 blockade [11].

## Conclusion

In summary, the metochalcone inhibited cell proliferation, migration, cell cycle progression, tumor growth, and induced senescence-associated secretory phenotype. These findings suggest that metochalcone is a potential candidate within chalcone family for combating breast and lung cancers via modulation of the JAK2/STAT3 signaling pathway and targeting STAT3 to induce senescence-associated secretory phenotype. These results broaden our understanding and provide a pharmacological approach for cancer senescence and senotherapy against carcinomas.

## Data Availability

The datasets used and/or analyzed during the current study are available from the corresponding author on reasonable request.
